# The effect of precordial lead displacement on ECG morphology

**DOI:** 10.1007/s11517-013-1115-9

**Published:** 2013-10-19

**Authors:** Michał Kania, Hervé Rix, Małgorzata Fereniec, Heriberto Zavala-Fernandez, Dariusz Janusek, Tomasz Mroczka, Günter Stix, Roman Maniewski

**Affiliations:** 1 Department of Biophysical Measurements and Imaging, Nalecz Institute of Biocybernetics and Biomedical Engineering, Polish Academy of Sciences, Trojdena 4, 02-109 Warsaw, Poland; 2 Laboratory of Informatics, Signals and Systems (I3S), National Center for Scientific Research (CNRS), University of Nice-Sophia Antipolis, Sophia Antipolis, France; 3 Department of Internal Medicine and Cardiology, Geriatric Center Wienerwald, Vienna, Austria; 4 Department of Cardiology, Medical University of Vienna, General Hospital of Vienna, Vienna, Austria

**Keywords:** Cardiac monitoring, Electrocardiogram, Body surface potential mapping, Precordial electrode displacement, Shape analysis

## Abstract

Inaccurate electrode placement and differences in inter-individual human anatomies can lead to misinterpretation of ECG examination. The aim of the study was to investigate the effect of precordial electrodes displacement on morphology of the ECG signal in a group of 60 patients with diagnosed cardiac disease. Shapes of ECG signals recorded from precordial leads were compared with signals interpolated at the points located at a distance up to 5 cm from lead location. Shape differences of the QRS and ST-T-U complexes were quantified using the distribution function method, correlation coefficient, root-mean-square error (RMSE), and normalized RMSE. The relative variability (RV) index was calculated to quantify inter-individual variability. ECG morphology changes were prominent in all shape parameters beyond 2 cm distance to precordial leads. Lead V_2_ was the most sensitive to displacement errors, followed by leads V_3_, V_1_, and V_4_, for which the direction of electrodes displacement plays a key role. No visible changes in ECG morphology were observed in leads V_5_ and V_6_, only scaling effect of signal amplitude. The RV ranged from 0.639 to 0.989. Distortions in ECG tracings increase with the distance from precordial lead, which are specific to chosen electrode, direction of displacement, and for ECG segment selected for calculations.

## Introduction

Electrocardiography is nowadays one of the most widely used diagnostic methods in screening tests for early detection of cardiac diseases. Examinations are noninvasive and have a large impact on clinical diagnosis and on further medical treatment. The ECG signals reflect the electrical activity of the heart muscle as it is sensed by electrodes placed on the body surface. In clinical practice, the most commonly used electrodes layout is 12-lead standard ECG system. Standard ECG has, however, limited sensitivity (30–70 %) and specificity (70–95 %) in detection of acute coronary syndromes [7]. To improve effectiveness of the ECG diagnostic, high-resolution measurement technique and body surface potential mapping (BSPM) were proposed [3, 29] and validated [5, 15, 31]. However, due to time-consuming procedure of large number ECG electrodes placement, the method is still not widely used in clinical practice.

There are many independent factors affecting the ECG examination results related to ECG measurement procedure and physiological inter-individual variability [27]. One of the main sources of mistakes is inaccurate ECG electrode placement in suggested anatomical landmarks, e.g., in proper intercostal spaces [14]. On the other hand, since differences in inter-individual human anatomies, the exact heart position in the thorax is never precisely known. Both factors, dependent and independent on medical staff, can cause the change of the distance between the electrodes and source of the signal in the heart as well as the solid angle at which outline of ventricular mass is seen from the body surface [34]. The displacements of the precordial electrodes located nearby the signal source have a greater influence on the ECG signal than shifts of the distant limb electrodes [20, 34]. Precordial electrodes need sometimes to be shifted to apply bandages, drains, and to undertake an echocardiographic study [19]. However, displacement of the ECG electrodes from determined ‘standard’ positions [14] can arise also from mistakes of medical staff [9, 18, 20, 23] as well as by patients at home who participate in ECG monitoring programs. A common mistake is placing V_1_ and V_2_ electrodes too high, in second or third intercostal space [20], which could result in superior misplacement of remaining precordial electrodes. Electrodes V_5_ and V_6_ are also placed frequently in the fifth intercostal space and not in the recommended parallel position to electrode V_4_ [14], which is usually not precisely positioned according to visual estimation of midclavicular line [21].

Thus, one important issue which should be taken into account is poor reproducibility of precordial lead placement in serial ECG recordings. Kerwin et al. [12] reported that correct lead positions with an error less than 1 cm were achieved by trained technicians only in case of 50 % of studied men and 20 % of studied women. They found that electrode placement error often was in the range of 2–3 cm, but occasionally reached even 6 cm.

A number of methods for controlling variation in chest electrodes’ position were suggested and validated. Soliman [30] recently proposed to add simple measurement of the distance from suprasternal notch to the V_1_–V_2_ position assuring the same position of electrodes between clinical trials. Herman et al. [9] invented a sliding ruler that facilitates correct lead placement and for documenting its position on the chest. Kerwin et al. [12] advise to use the grid printed on non-stretchable material to record and later on, if needed, to relocate wrong positioned electrodes. Unlikely, all methods, even the simplest, are not accepted by clinicians, meaning that ECG electrodes are often placed not precise according to subjective visual inspection.

The analysis of ECG signals recorded from misplaced electrodes can lead to misinterpretation or even to significant diagnostic errors like incorrect recognition of anterior infarction, anteroseptal infarction, ventricular hypertrophy [9, 14], false diagnosis of ischemia, or Brugada syndrome [16, 24]. Bond et al. have shown that incorrect electrode placement could lead to wrong diagnosis in 17–24 % of patients [1]. Precordial electrode displacement could cause wrong diagnosis made by human expert as well as by computer-based analysis [26].

The aim of this study was to investigate in detail the effect of displacement of the precordial ECG electrodes on the morphology of the recorded multilead high-resolution ECG signals, in particular, to answer the questions what kind of changes in the recorded ECG signal could be expected while moving the electrode in any direction at a short distance (up to 5 cm) and which precordial ECG leads are most sensitive to electrodes displacement.

## Methods

### Measurement and processing of ECG signal

**Fig. 1:**
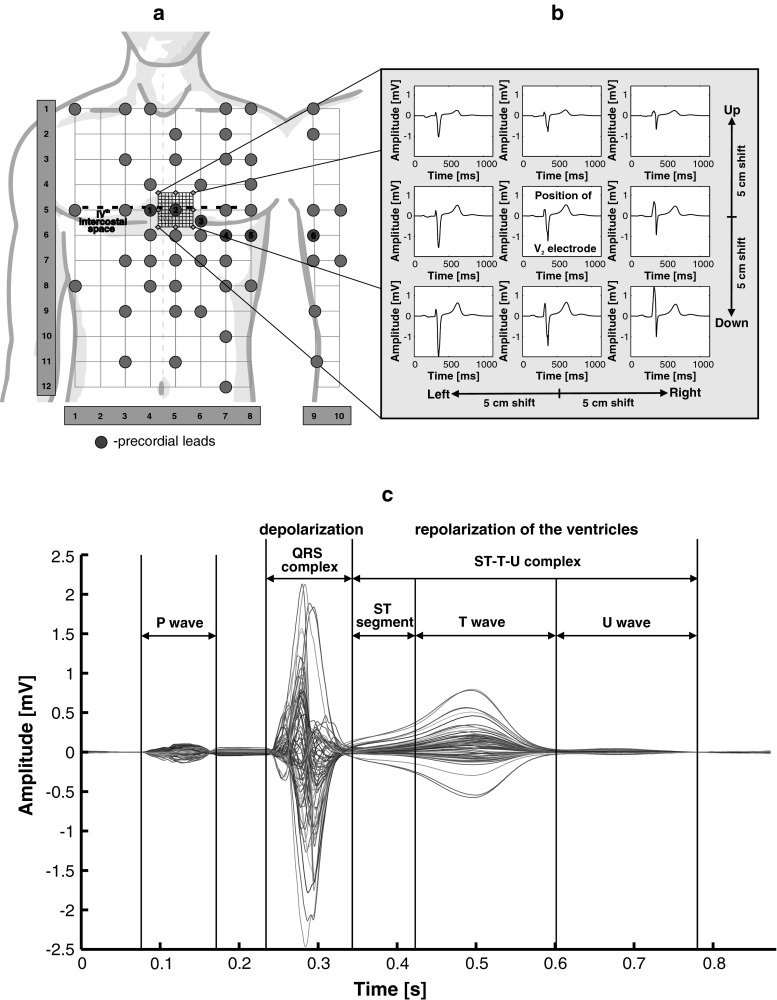
**a** BSPM measurement layout and location grid of interpolated ECG signal around lead V_2_. The location grid contains 11 × 11 nodes spaced apart by 1 cm. ECG signals were interpolated based on data sensed by BSPM electrodes *circle*. **b** ECG signals obtained by interpolation for nodes of grid marked by *diamond* in *panel A*. **c** Time-aligned-averaged superimposed ECG signals measured from 64 locations on thorax surface

This study was performed on a group of 60 men with diagnosed cardiac pathology. The patients’ age ranged from 38 to 83 years. Basic statistic data of the studied group are presented in Table 1. Examinations were carried out in General Hospital of Medical University of Vienna (Austria) using a high-resolution ECG measurement system (Biosemi). The 64 active electrodes were placed on the body surface according to modified Amsterdam lead system [4, 29]. The electrodes’ positions on the chest surface used in this study are shown in Fig. 1a.

**Table 1:** Basic data of studied group

Mean ± SD						Number of patients with specified pathology				
Age (years)	Height (cm)	Weight (kg)	BMI (kg/m^2^)	dSU (cm)	dC (cm)	MI	CAD	ICD	BBB	DCM
62.74 ± 11.74	174.75 ± 7.08	87.49 ± 12.86	28.62 ± 3.59	110.27 ± 7.53	40.27 ± 3.03	39	46	33	7	2

*MI* myocardial infarction, *BMI* body mass index, *CAD* coronary artery disease, *BBB* bundle branch block, *DCM* dilated cardiomyopathy, *ICD* implantable cardioverter defibrillator, *dSU* distance between sternal notch and umbilicus, *dC* circumference of the thorax at level of IV intercostial space, *SD* standard deviation

The multi-lead high-resolution ECG was recorded for 5 min with 4,096 Hz sampling frequency and digitized with 24-bit amplitude resolution while subject was at rest in supine position. The study protocol was approved by an institutional ethical committee in accordance with Declaration of Helsinki, and informed consent was obtained from each patient. Data were band-pass filtered with cutoff frequencies 0.05, 250 Hz. Then, linear baseline wander estimation and removal procedure were applied (U-P segment used as isoelectric line). The cross-correlation method for beats alignment was used, and then, signal averaging in time was performed. ECG characteristic points in averaged signals were first automatically detected as in [6]. Then, the result for each subject was visually examined and edited based on the view of time-aligned superimposed heartbeats (Fig. 1c).

### Evaluation of ECG signals in close distance to precordial electrodes

The position of ECG signal estimation points in close distance (1–5 cm) from precordial electrodes (V_1_–V_6_) was determined for each subject as shown in Fig. 1 for electrode V_2_. Then, ECG signals were interpolated [25] in 11 × 11 coordinates of rectangular grid centered on selected precordial lead position (Fig. 1a). Shapes of estimated signals were compared with shape of reference signal recorded in precordial lead location. The analysis was performed for depolarization (QRS complex) and repolarization (ST-T-U segment) phases of cardiac cycle (Fig. 1c).

In order to precisely evaluate changes in ECG signal morphology caused by a shift of ECG electrodes, the distribution function method (DFM) proposed by Rix and Malengé [22] was used. It was shown that DFM is independent of amplitude and timescale variation of a signal, assessing the real shape changes and provides valuable information for cardiac diagnosis [5, 13].

Let *s*_0_(*t*) be a reference signal and *s*_*j*_(*t*) a signal to compare, whose supports are included in time interval [0, T]. If *s*_0_(*t*) and *s*_*j*_(*t*) are equal in shape, signal *s*_0_(*t*) can be derived from *s*_*j*_(*t*) through increasing affine functions:

1 $$s_{0} \left( t \right) = k_{j} s_{j} \left( {a_{j} t + t_{j} } \right) + c_{j} ; \quad a_{j} > 0, \, k_{j} > 0 ,$$

where *k*_*j*_, *a*_*j*_, *t*_*j*_, *c*_*j*_ are, respectively, magnitude coefficient, scale coefficient, delay, and offset.

Since ECG baseline drift is subtracted in preprocessing stage, *c*_*j*_ value can be omitted and Eq. (1) is rewritten as follows:

2 $$s_{0} \left( t \right) = k_{j} s_{j} \left( {a_{j} t + t_{j} } \right); \quad a_{j} > 0,\, k_{j} > 0,$$

which corresponds to the following:

3 $$s_{j} \left( {t'} \right) = k_{j}^{'} s_{0} \left( {\frac{{t^{'} - t_{j} }}{{a_{j} }}} \right) ,\quad {\text{where}}\,t^{'} = a_{j} t + t_{j} \quad {\text{and}}\quad k_{j}^{'} = \frac{1}{{k_{j} }}.$$

Assuming *s*_0_(*t*) and *s*_*j*_(*t*) are positive signals, the shape difference between two ECG waves was characterized by the function *φ*(*t*) defined by the relation:

4 $$S_{j} \left( t \right) = S_{0} \left( {\varphi \left( t \right)} \right) \quad {\text{i}} . {\text{e}}.\,\varphi = S_{0}^{ - 1} \circ S_{j} ,$$

where *S*_*j*_ *(t)* and *S*_*0*_ *(t)* are the normalized integral functions of *s*_*j*_ *(t)* and *s*_*0*_ *(t)*, respectively, rising from 0 to 1 on the signal support:

5 $$S_{j} \left( t \right) = \frac{{\mathop \int \nolimits_{0}^{t} s_{j} \left( \tau \right){\text{d}}\tau }}{{\mathop \int \nolimits_{0}^{T} s_{j} \left( \tau \right){\text{d}}\tau }} ,\quad   S_{0} \left( t \right) = \frac{{\mathop \int \nolimits_{0}^{t} s_{0} \left( \tau \right){\text{d}}\tau }}{{\mathop \int \nolimits_{0}^{T} s_{0} \left( \tau \right){\text{d}}\tau }},$$

The interval [0, 1] is divided by *M* equidistant values *y*_*i*_: 0 < *y*_*i*_ < 1, *i* = 1 to *M*. Solving the equation by linear interpolation: *y*_*i*_ = *S*_0_(*t*’_*i*_) = *S*_*j*_(*t*_*i*_) gives a set of couples (*t*_*i*_, *t*’_*i*_) linked by *t*’_*i*_ = *φ*(*t*_*i*_).

If signals $s_{0}$ and $s_{j}$ have the same shape, then points (*t*_*i*_, *t*’_*i*_) are on a straight line, corresponding to an affine function $\varphi$. The departure from linearity was quantitated through calculation of *Δ* parameter, which is the root-mean-square error (RMSE) between the set of points (*t*_*i*_, *t*’_*i*_) and the least-mean-square line

6 $$y\left( t \right) = \alpha t + \beta$$

fitted on $$\varphi (t_{i} )$$ [5]:

7 $$\Updelta = \sqrt {\frac{1}{M}\sum\nolimits_{i = 1}^{i = M} {\left( {\varphi \left( {ti} \right) - y(ti} \right)^{2} } }$$

where *α*—regression coefficient measuring the timescale change of *s*_*j*_(*t*) compared to *s*_0_(*t)*. The meaning of *Δ* parameter is that it describes the real ECG morphology changes between a pair of two signals $s_{0}$ and $s_{j}$ omitting the scaling effect, i.e., stretching or compressing of ECG waves either in amplitude or in time. Parameter *Δ* close to zero means that the main waveform pattern does not change. In contrast, *Δ* greater than zero indicates appearance or disappearance of ECG components (e.g., QRS complex change its shape to rSr’ pattern). The coefficient *α* in Eq. 6 indicates if and how much reference signal *s*_0_ is stretched (*α* > 1) or shrunken (*α* < 1) in time. Later on, it will refer to scaling effect of ECG signal in time.

The Pearson correlation coefficient (*R*) and RMSE between reference signal *s*_0_(*t*) and signals *s*_*j*_(*t*) were also calculated to compare obtained results with those available in the literature. For an ECG signal measured in particular precordial electrode position and ECG signal observed at a given distance from that location, the RMSE was defined as follows:

8 $${\text{RMSE}} = \sqrt {\frac{1}{N}\sum\nolimits_{i = 1}^{i = N} {\left( {s_{0} (ti) - s_{j} (ti)} \right)^{2} } } ,$$

where *N*—number of samples in the averaged ECG signal, *s*_0_(*ti*)—the amplitude of measured ECG signal in *i*th sample (reference signal), *s*_*j*_(*ti*)—the amplitude of interpolated ECG signal *s*_*j*_ in *i*th sample.

RMSE parameter was normalized to range of the observed data in order to minimize the effect of inter-individual differences in ECG signal magnitude on mean RMSE values computed in studied group.

9 $${\text{NRMSE}} = \frac{\text{RMSE}}{{S_{{{\text{obs}},\hbox{max} }} - S_{{{\text{obs}},\hbox{min} }} }}.$$

The relative variability index (RV) defined by Hoekema et al. [10] was calculated to quantitate inter-individual variability of measured body surface potentials. QRS and ST-T-U complexes were normalized to 800 samples. RV index for 11 × 11 ECG data points (Fig. 1a), for a given precordial lead, was defined as the averaged variances over all subjects, for each grid node, and each time instant divided by the overall power of recorded signal:

10 $${\text{RV}} = \sqrt {\frac{{\frac{1}{L \cdot T}\mathop \sum \nolimits_{l = 1}^{l = L} \mathop \sum \nolimits_{t = 1}^{T} \frac{1}{K}\mathop \sum \nolimits_{i = 1}^{K} \left( {V_{i,l,t} - \overline{V}_{l,t} } \right)^{2} }}{{\frac{1}{L \cdot T \cdot K}\mathop \sum \nolimits_{l = 1}^{L} \mathop \sum \nolimits_{t = 1}^{T} \mathop \sum \nolimits_{i = 1}^{K} V_{i,l,t}^{2} }}} ,$$

where *V*_*i,l,t*_ is the amplitude of ECG signal in grid node *l*, in time *t,* for subject *i*; $\overline{V}_{l,t}$ is the mean signal over all subjects in grid node *l*, in time *t*; *L* is the number of nodes (*L* = 121) in data grid; *T* is the number of time instants (*T* = 800); *K* is the number of studied subjects (*K* = 60). The relation is equivalent to ratio of standard deviation to root-mean-square value of all the measured ECG signals.

Additional analysis was performed in order to distinguish ECG changes caused by electrode displacement from physiological variability of ECG shape in time. Five sets of precordial ECG signals (V_1_–V_6_) averaged in time were computed separately for each subject from five consecutive 1-min recordings. ECG signals from the first set were compared with corresponding signals from other sets. The variability of ECG signal morphology in time was quantified by shape difference parameters.

## Results

**Fig. 2:**
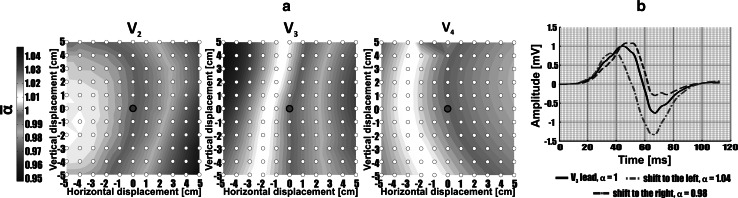
**a** Body surface distributions of $\overline{\alpha }$ parameter quantifying the scale change of QRS complex in time domain for positions of interpolated ECG signals in relationship to precordial leads location (marked by *red dot* in the *center* of a map), **b** averaged QRS complex from lead V_3_ for one of studied patient compared to QRS complexes interpolated in the 5 cm distance (to the *left*/*right*) from V_3_ position (colour figure online)

**Fig. 3:**
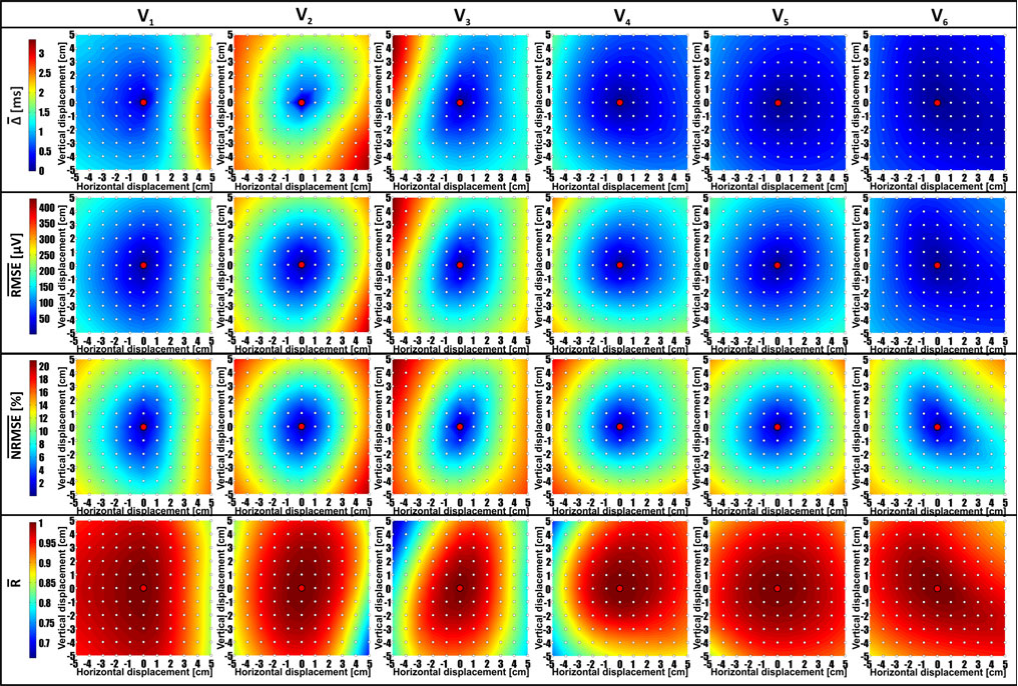
Body surface distributions of mean values of ECG shape descriptors *Δ*, RMSE, NRMSE, and *R* computed for QRS complex in the studied patients’ group. Distributions of parameter values on the body surface quantitating the shape difference between QRS complex recorded in the standard position of selected precordial electrode (marked by *red dot* in the *center* of each map) and QRS signal interpolated in a given distance from the correct electrode position. Maps for each parameter are presented in separate row. Subsets of *SDM* for a given precordial electrode are shown in subsequent columns. The distance between neighboring interpolation points corresponds to 1 cm shift of the standard precordial electrode (colour figure online)

**Fig. 4:**
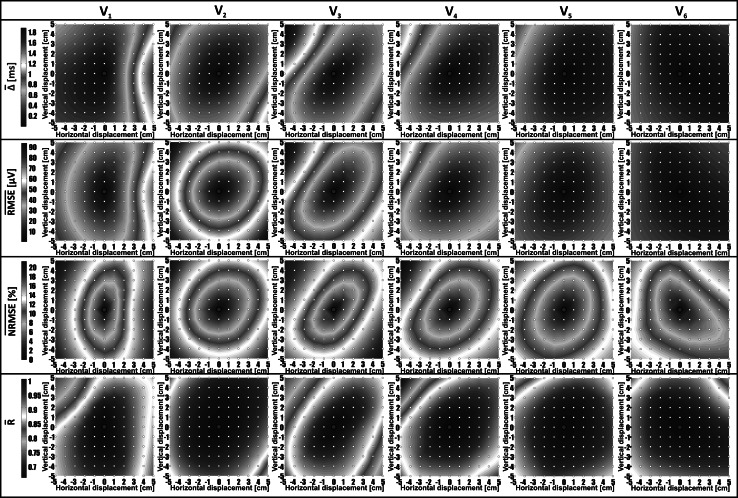
Body surface distributions of $\overline{\Updelta }$, $\overline{\text{RMSE}}$, $\overline{\text{NRMSE}}$, and $\overline{R}$ parameters computed for ST-T-U segment in the studied patients group. Distributions of parameter values on the body surface quantifying the shape difference between ST-T-U segment recorded in the standard position of selected precordial electrode (marked by *red dot* in the *center* of each map) and ST-T-U segment interpolated in a given distance from correct electrode position. Maps for each parameter are presented in a separate row. The subsets of SDM for a given precordial electrode are shown in subsequent columns. The distance between neighboring interpolation points corresponds to 1 cm shift of the standard precordial lead (colour figure online)

Distributions of computed mean parameters around precordial electrodes’ positions, called ‘shape difference maps (SDM)’, are presented in Figs. 2, 3, and 4. Mean values of shape difference descriptors of QRS complex and ST-T-U segment were calculated in the studied group and are denoted by $\overline{\alpha }$, $\overline{\Updelta }$, $\overline{\text{RMSE}}$, $\overline{\text{NRMSE}}$, and $\overline{R}$. Color scales of SDM were normalized. The red (blue) color corresponds to the maximum (minimum) value for a given shape parameter. The neighboring interpolation points marked by white dots are separated by 1 cm.

The distribution of $\overline{\alpha }$ (Eq. 6) for selected distances to the standard V_2_, V_3_, and V_4_ electrodes calculated for QRS complex is presented in Fig. 2a. Remaining maps of parameter $\overline{\alpha }$ computed for other precordial electrodes are not shown due to negligible observed changes ($\overline{\alpha }$ values were in the range from 0.994 ± 0.129 to 1.010 ± 0.136.) Scaling effect of ST-T-U segment in time for all precordial leads was not observed. ($\overline{\alpha }$ values varied from 0.988 ± 0.129 to 1.002 ± 0.131.) Averaged QRS complexes for one case are presented in Fig. 2b. The 5 cm displacement of V_3_ electrode in direction to V_2_ position causes stretching of QRS complex in time by factor α = 1.04, while the V_3_ shift by 5 cm in opposite direction causes shrinking of QRS complex in time by factor α = 0.98.

**Fig. 5:**
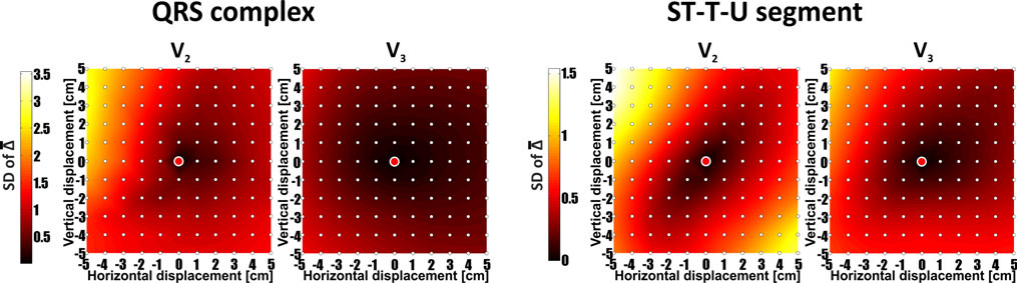
Distribution of standard deviations (SD) from a $\overline{\Updelta }$ parameter computed in QRS complex and ST-T-U segment for a given distance from precordial electrodes V_2_ and V_3_ (positioned in the center of the map)

The representative maps of standard deviations from mean shape difference descriptors *Δ* are shown in Fig. 5. SD values for QRS complex were in the range from 0 to 0.15 for $\overline{\alpha }$, from 0 to 3.5 for $\overline{\Updelta }$, from 0 to 250 μV for $\overline{\text{RMSE}}$, from 0 to 10 % for $\overline{\text{NRMSE}}$, and from 0 to 0.4 for $\overline{R}$. The observed SDs in case of ST-T-U segment varied from 0.13 to 0.14 for $\overline{\alpha }$, from 0 to 1.5 for $\overline{\Updelta }$, from 0 to 70 μV for $\overline{\text{RMSE}}$, from 0 to 10 % for $\overline{\text{NRMSE}}$, and from 0 to 0.5 for $\overline{R}$.

Table 2 presents the numerical values of $\overline{\Updelta }$, $\overline{\text{RMSE}}$, $\overline{\text{NRMSE}}$, and $\overline{R}$ parameters quantifying the ECG shape changes due to electrode shifts at 1 and 5 cm. Notice that the largest values are marked in bold font.

**Table 2:** Maximal observed changes of ECG morphology at 1 and 5 cm distance from precordial electrode positions

Parameter	Cardiac phase	Precordial electrode					
		V_1_	V_2_	V_3_	V_4_	V_5_	V_6_
1 cm							
$\overline{\Updelta }$ (ms)	QRS complex	0.7 ± 0.4	**0.9** **±** **1.9**	0.5 ± 0.8	0.2 ± 0.1	0.1 ± 0.1	0.1 ± 0.1
	ST-T-U segment	**0.3** **±** **0.2**	0.2 ± 0.2	0.2 ± 0.2	0.2 ± 0.1	0.1 ± 0.1	0.1 ± 0.1
$\overline{\text{RMSE}}$ (μV)	QRS complex	41 ± 22	48 ± 28	**67** **±** **35**	41 ± 23	28 ± 13	21 ± 10
	ST-T-U segment	13 ± 9	11 ± 8	**13** **±** **9**	8 ± 5	5 ± 4	4 ± 3
$\overline{\text{NRMSE}}$ (%)	QRS complex	4 ± 2	3 ± 2	**4** **±** **3**	3 ± 2	2 ± 1	3 ± 2
	ST-T-U segment	**6** **±** **5**	3 ± 3	5 ± 4	4 ± 3	3 ± 3	3 ± 2
$\overline{R}$	QRS complex	**0.99** **±** **0.03**	0.99 ± 0.01	0.99 ± 0.01	0.99 ± 0.01	0.99 ± 0.01	0.99 ± 0.01
	ST-T-U segment	**0.98** **±** **0.07**	0.99 ± 0.01	0.99 ± 0.03	0.99 ± 0.02	0.99 ± 0.03	0.99 ± 0.01
5 cm							
$\overline{\Updelta }$ **(**ms)	QRS complex	**2.7** **±** **2.7**	2.5 ± 3.3	2.6 ± 2.4	1.2 ± 0.9	0.8 ± 0.4	0.7 ± 0.4
	ST-T-U segment	**1.6** **±** **1.4**	0.7 ± 0.8	1.2 ± 1.0	0.9 ± 0.8	0.6 ± 0.5	0.6 ± 0.5
$\overline{\text{RMSE}}$ **(**μV)	QRS complex	235 ± 126	292 ± 158	**344** **±** **192**	235 ± 140	145 ± 65	125 ± 63
	ST-T-U segment	**73** **±** **53**	69 ± 52	63 ± 44	46 ± 33	27 ± 19	23 ± 19
$\overline{\text{NRMSE}}$ (%)	QRS complex	15 ± 8	16 ± 8	**18** **±** **9**	14 ± 10	12 ± 4	12 ± 5
	ST-T-U segment	**19** **±** **11**	16 ± 9	16 ± 10	15 ± 9	13 ± 6	14 ± 7
$\overline{R}$	QRS complex	0.85 ± 0.28	0.83 ± 0.28	**0.78** **±** **0.28**	0.82 ± 0.32	0.94 ± 0.13	0.93 ± 0.10
	ST-T-U segment	0.85 ± 0.33	0.90 ± 0.24	**0.82** **±** **0.31**	0.86 ± 0.32	0.90 ± 0.22	0.91 ± 0.22

Bold font highlights the largest ($\overline{\Updelta }$, $\overline{\text{RMSE}}$, $\overline{\text{NRMSE}}$) and smallest ($\overline{R}$) values ± SD of ECG shape difference descriptors

Obtained results from analysis of time variability of ECG signal shape in precordial leads are summarized in Table 3. The mean values ± SD of $\overline{\Updelta }$, $\overline{\text{RMSE}}$, $\overline{\text{NRMSE}}$, and $\overline{R}$ were shown. The values highlighted in bold were used as threshold to decide about significance of shape changes due to electrode displacement, and they represented the largest observed ECG signal changes in time.

**Table 3:** Time variability of ECG morphology

Parameter	Cardiac phase	Precordial electrode					
		V_1_	V_2_	V_3_	V_4_	V_5_	V_6_
$\overline{\Updelta }$ (ms)	QRS complex	0.34 ± 0.26	**1.15** **±** **0.71**	0.30 ± 0.18	0.08 ± 0.06	0.07 ± 0.05	0.05 ± 0.03
	ST-T-U segment	0.11 ± 0.10	**0.17** **±** **0.13**	0.16 ± 0.12	0.12 ± 0.08	0.09 ± 0.07	0.05 ± 0.04
$\overline{\text{RMSE}}$ (μV)	QRS complex	18 ± 13	**25** **±** **17**	23 ± 15	20 ± 14	18 ± 12	11 ± 79
	ST-T-U segment	8 ± 7	**12** **±** **9**	9 ± 7	7 ± 5	5 ± 4	3 ± 2
$\overline{\text{NRMSE}}$ (%)	QRS complex	1.1 ± 0.8	1.2 ± 0.9	1.2 ± 0.8	1.2 ± 0.8	**1.6** **±** **1.1**	1.6 ± 1.1
	ST-T-U segment	3.1 ± 2.5	2.4 ± 1.8	4.1 ± 3.0	4.2 ± 2.8	**4.4** **±** **3**	4 ± 3
$\overline{R}$	QRS complex	0.999 ± 0.001	0.998 ± .001	0.999 ± .001	0.999 ± .001	**0.997** **±** **.002**	0.998 ± .001
	ST-T-U segment	0.996 ± 0.005	0.997 ± .003	**0.978** **±** **.017**	0.988 ± .011	0.984 ± .016	0.990 ± .001

Bold font highlights the largest ($\overline{\Updelta }$, $\overline{\text{RMSE}}$, $\overline{\text{NRMSE}}$) and smallest ($\overline{R}$) values ± SD of ECG shape difference descriptors for a given cardiac phase

## Discussion

In obtained results, distinct changes of ECG morphology were observed when precordial electrodes were displaced. In Fig. 1b, the example of ECG signal recorded from lead V_2_ at correct and displaced position is presented. The changes in ECG signal morphology are clearly visible while moving the electrodes from their correct positions.

Distributions of all evaluated parameters ($\overline{\Updelta }$, $\overline{\text{RMSE}}$,$\overline{\text{NRMSE}}$, and $\overline{R}$) in relation to electrode shift in a given direction were coincident. The dispersion of the signal morphology was growing with the increase in the distance from the reference point (Tables 2 and 3). That shape disparity was observed both in the mean shape difference values and its standard deviation values (shape difference maps in Figs. 3 and 4 versus corresponding SD maps presented in Fig. 5). The $\overline{R}$ parameter was less sensitive to changes of precordial electrode positions compared with other calculated parameters.

The shift up to 1 cm from precordial leads in any direction has the negligible impact on the ECG morphology (Table 2). Mean values of shape difference descriptors were in the range of physiological variability of ECG signal morphology in time (Table 2). These results are in agreement with the outcome of study presented by Szakolczai et al. [32] where the mean morphology change of a whole cardiac cycle observed in V_2_ electrode shifted by 1 cm in vertical and horizontal directions was *R* = 0.98 ± 0.04, RMSE = 67 ± 28 μV. In our analysis, more prominent morphology changes of ECG waves were found for electrode displacements of 2 cm or higher. This supports the results of simulation study performed by Turzova et al. [33] where the mean relative error between body surface potential maps estimated in standard and vertically shifted electrodes’ positions remained less than 5 % up to ±2 cm shift. Beyond the threshold value set to 2 cm differences in BSPM steeply increased [33].

In our study, mean values of RMSE ± SD calculated for ST-T-U segment were in the range from 12 ± 9 to 73 ± 53 μV for the vertical shift of electrodes by 5 cm. Finlay et al. (2010) reported similar trend of RMSE values changes in response to vertical shift (0.5–5 cm) of precordial electrodes. In their work, the median of RMSEs calculated for ST-T segment in 5 cm distance from correct precordial electrodes’ positions ranged between 30 μV and 130 μV [8]. Lower values of RMSE parameter obtained in our study may result from longer segment of ECG signal used for calculations as well as influence of inter-individual differences in measured signal amplitude.

A degree of morphology changes depends on ECG lead, shift magnitude, direction of displacement, and on the ECG segment selected to analysis. We found that leads V_2_ and V_3_ are the most sensitive to displacement (Figs. 3, 4). For example, a 5 cm left displacement of V_3_, in direction to V_2_ position, causes significant changes in QRS shape and ST-T-U curves (Figs. 3, 4). This is partially in agreement with the results obtained by Bond et al. [1]. They report the V_2_ as the most affected, but they indicated electrode V_3_ together with V_5_ and V_6_ as the least sensitive to displacement. It could be due to the fact that they compared ECG morphology changes in electrodes placed in one specific nonstandard ECG leads arrangement.

Besides the shift magnitude, directions of precordial lead displacement have significant impact on ECG signal morphology (Fig. 1b). Lead V_1_ is more sensitive to horizontal than vertical displacement, and ECG morphology changes more prominently while shifting electrode toward V_2_ position. Negligible differences in $\overline{\Updelta }$ parameter values caused by the shift of the V_1_ electrode in vertical directions, with observed differences in $\overline{\text{NRMSE}}$ values (Figs. 3, 4), suggest that ECG signal is changing mainly due to change in the amplitude without significant changes in the main waveform pattern (amplitude scaling effect).

The V_2_ displacement affects more the QRS complex morphology than ST-T-U segment (less prominent changes of $\overline{\Updelta }$ parameter shown in Fig. 4 in comparison with maps of $\overline{\Updelta }$ parameter presented in Fig. 3). Displacement of the electrode V_2_ in direction to V_3_ causes slight shrinking of QRS complex in time (decrease in the values of $\overline{\alpha }$ parameter shown in Fig. 2). There were no observed scaling effect in time for ST-T-U wave (reported by $\overline{\alpha }$ parameter), as well as only slight changes in $\overline{\Updelta }$ parameter observed in the distance of 5 cm from V_2_ in direction to V_3_ electrode with more evident changes in $\overline{\text{NRMSE}}$ values. The changes of ST-T-U segment are probably more connected to effect of signal amplitude scaling than to changes in the main waveform pattern.

Displacement of V_3_ and V_4_ electrodes in direction to V_2_ position is crucial for QRS complex morphology. Moving electrodes in this direction causes slight stretching of QRS wave as shown in Fig. 2. Shifting V_3_ and V_4_ electrodes along a left diagonal more significantly affects ST-T-U segment than displacement along right diagonal as observed in the SDM (Figs. 3, 4).

Changes observed in maps of $\overline{\text{NRMSE}}$ parameter for V_5_ and V_6_ are not confirmed by the values of $\overline{\Updelta }$, $\overline{\alpha }$, $\overline{\text{RMSE}}$, and $\overline{R}$ parameters. These suggest that differences in ECG signal in V_5_ and V_6_ are more connected to ECG amplitude scaling than to morphology changes.

High values of inter-individual relative variability were observed in the precordium from 0.639 (V_6_) to 0.886 (V_3_) for QRS complex and from 0.693 (V_2_) to 0.989 (V_5_) for ST-T-U wave. In anterior leads V_2_ and V_3_, higher values of RV were found for QRS complex than for ST-T-U segment. In the remaining precordial leads, higher RV was observed for ST-T-U segment in comparison with QRS complex. Hoekema computed relative variability index for 25 healthy subjects and found that the RV of QRS complex in close distance (up to 3.8 cm in the vertical direction) to V_2_ electrode was 0.503 [10].

The large variability of measured ECG signal depends on the anatomical differences between studied patients, e.g., different position and orientation of the heart in the chest or different torso geometry. The high inter-individual variability found in ECG signal affects the mean values of non-normalized shape difference parameters like root-mean-square error. Therefore, more appropriate ECG parameters for subjects’ comparison are normalized descriptors like $\overline{\Updelta }$, $\overline{\text{NRMSE}}$, and $\overline{R}$.

There is an accepted rule of ECG electrodes’ positioning according to the anatomical landmarks like intercostal spaces. However, in women, precordial electrodes are often positioned under the breast what could be the reason for unsatisfactory reproducibility. It comes from concerns that the ECG amplitudes are attenuated substantially by the breast tissue. Rautaharju et al. [21] showed that breast size accounted only for <1 % of ECG amplitude variations. They recommended placing electrodes on the breast in standardized positions and propose to use special device to deal with not trivial task of electrodes’ positioning, especially in women with large breast tissue. In the present study, we examined only male patients, but in light of mentioned study, our results could be also valid for assessment of electrodes displacement effect in women.

Experimental studies [10] have shown that heart in the chest may be situated in the frontal plane at a distance up to 3 cm from the position of precordial electrode V_2_ on the chest. This difference in distance between surface sensor and the source inside the torso caused change in distribution and amplitudes of heart potentials measured on the body surface [17, 34]. Thus, placing electrodes even in accordance with the existing standard does not ensure that for all subjects, they are located in the same relation to the heart position. The use of body surface potential mapping might be considered to avoid not precise positioning of ECG electrodes in relation to heart location. This was already pointed out in BSPM studies showing a more complete view of the electrical activity of the heart and valuable diagnostic information not visible in standard 12-lead ECG [2, 11, 15].

Our study demonstrated complexity of the problem of ECG morphology distortion as a consequence of precordial electrodes displacements. We focused on detailed description of the influence of precordial electrodes displacement in any direction on ECG morphology in QRS and ST-T-U segments. Whether observed changes have significant effect on clinical diagnosis still remains under question and need clinically oriented studies. It was already shown that shifts of precordial leads by 2 cm can result in altered R wave progression and shift in the precordial transition zone, respectively, in 20 and 75 % of patients [9] as well as altered QRS complex and T wave [12] leading to misinterpretation regarding anteroseptal infarction and ventricular hypertrophy [9], or false statements about appearance of myocardial ischemia or right bundle branch block [12]. The analysis of ECG signals recorded from vertically displaced V_1_ and V_2_ leads could also give false impression of Brugada syndrome [16]. Schijvenaars et al. [26, 28] studied the relation of horizontal simultaneous displacement of V_1_–V_3_ leads on the changes of computer-based diagnosis concerning MI and left ventricular hypertrophy (LVH). In their experiment, V_1_ was shifted rightward up to 3 cm, V_2_ was shifted leftward up to 3 cm, and V_3_ was moved leftward half of this distance. Important classification changes caused by such lead displacements were observed in less than 1.5 % cases (for MI) and less than 1 % (for LVH). Our results showed that in such constructed experiment, the most affected lead in the sense of morphology changes could be only lead V_2_, where the changes of $\overline{\Updelta }$ were visible in contrast to lead V_1_ and V_2_ (Fig. 3). The worst effect could be expected if the leads V_1_ and V_2_ will be shifted in direction to each other.

The high inter-individual variability of ECG amplitude in studied patients’ group suggests important influence of specific human anatomy on measured ECG signals. Therefore, non-normalized ECG parameters like RMSE should be avoided for comparison of data from different subjects. The $\overline{\Updelta }$ parameter calculated using the DFM gives additional information about the shape changes of ECG signal being more sensitive to real morphology changes. Multiparameter analysis performed gives complete view, showing time and amplitude’s scaling effects of ECG signal due to electrode displacements. Obtained results may help to choose alternative locations of precordial electrodes when there is need to expose the space on the body surface for other diagnostic procedures.
